# Completed Suicide by Firearm in an Individual With the Agrammatic Variant of Primary Progressive Aphasia: Case Report

**DOI:** 10.3389/fneur.2022.828155

**Published:** 2022-03-15

**Authors:** Deepal Patel, Shaun Andersen, Kyler Smith, Aaron Ritter

**Affiliations:** ^1^Kirk Kerkorian School of Medicine, Las Vegas, NV, United States; ^2^University of Nevada, Reno, NV, United States; ^3^Cleveland Clinic Lou Ruvo Center for Brain Health, Las Vegas, NV, United States

**Keywords:** suicide, primary progressive aphasia, dementia, gun safety, Frontotemporal dementia (FTD)

## Abstract

The agrammatic or nonfluent variant of Primary Progressive Aphasia (nfvPPA) is a form of Frontotemporal Dementia (FTD) that is characterized by progressive language dysfunction, poor sentence construction, and low verbal fluency. Individuals with nfvPPA have intact insight into their decline, which may manifest as frustration and hopelessness, and show signs of impulsivity and disinhibition. Little is known about suicide risk in this patient population. Here we describe a case of an 84 year-old male with nfvPPA who, over the course of his care, experienced a decline in language and motoric functioning which coincided with increasing irritability and impulsivity. Despite this significant decline, he denied depressive symptoms or showed any suicidal tendencies, and he seemed to be looking forward to future events. His suicide, committed with a handgun during what appeared to be a rather innocuous trip to the garage, came as a significant shock to his spouse, family, and his clinical care team. To our knowledge, this is the first reported case of completed suicide in a patient with the nfvPPA subtype of FTD. Though this patient demonstrated demographic risk factors for suicide (advanced age, retired military veteran with easy access to firearms) there is a lack of data regarding how FTD may have contributed. Retained insight especially seems to be a risk factor for suicide across all forms of dementia. Impulsivity may be key when considering suicidality amongst FTD patients. Additionally, this case demonstrates the importance of addressing gun safety as there are few guidelines around gun ownership in this patient population.

## Description

With an estimated one million deaths each year, suicide remains an unmet global health crisis (1). This is especially relevant to those over the age of 70 who have the highest completion rates (2). Identification and prevention of suicide may be even more difficult in elderly populations who may show fewer warning signs than younger individuals (3). Known risk factors for suicide in older individuals include depression (4), social isolation (5), alcoholism (6), retirement (7), and medical illness/physical disability (8). These risk factors have become even more important during the COVID-19 pandemic, which has disproportionately affected the elderly (9, 10). New approaches to suicide research are attempting to develop integrated models that incorporate the myriad of complex neurobiological, psychological, and social factors that may contribute to suicidal behavior in both younger and older populations (11).

Dementia is a unique problem amongst the elderly and becomes highly prevalent after the age of 65 (12). Over the past three decades of dementia research, our understanding of the complex relationship between suicide and dementia has continued to evolve (2). Historically, the risk of suicide was thought to be similar to that in age-matched, non-demented populations (13). This research was largely conducted in “all cause” dementia populations, which can reliably be assumed to be composed largely of individuals with Alzheimer's disease (AD) (14). Certain phenotypic characteristics of AD dementia, however, may protect against suicide including poor insight and high levels of amnesia, especially as the disease progresses (4, 15). These protective features may be lacking in other forms of dementia. New data suggests that risk for suicide may be significantly higher in several non-AD forms of dementia including Huntington's disease (HD) and Frontotemporal dementia (FTD) (16–18).

Primary progressive aphasia (PPA) is a form of FTD that is characterized by progressive language dysfunction (19). Current guidelines define three subtypes of PPA based on clinical features and supporting biomarkers (20). The agrammatic or nonfluent variant (nfvPPA) of PPA is characterized by poor sentence construction, low verbal fluency, and effortful speech, thus often resembling Broca's aphasia. Many individuals with nfvPPA progress to a syndrome resembling Progressive Supranuclear Palsy (PSP) or Corticobasal Syndrome (CBD) and may develop behavioral symptoms such as impulsivity, disinhibition, and apathy, which may become very severe and functionally disabling (21). In stark contrast to patient with AD who are often unbothered or deny the existence of any decline, many patients with nfvPPA are acutely aware and can be extremely bothered by their cognitive deficits. The neuropsychiatry of PPA has not been extensively studied and little is known about suicide risk in PPA (22). Here we describe what we believe to be the first case report of suicide in the nfvPPA subtype of FTD.

## Case Report

**Figure 1:**
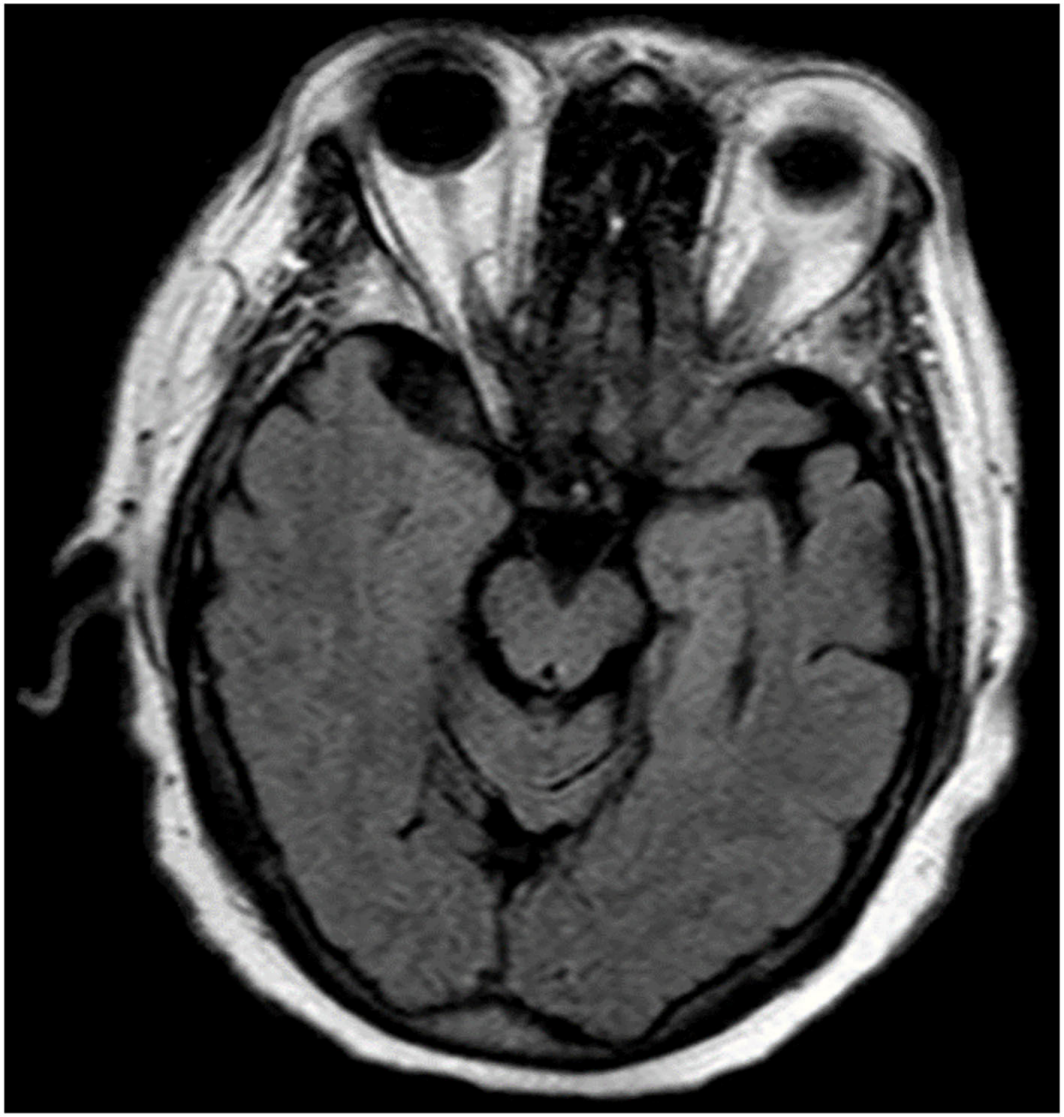
The patient's magnetic resonance imaging revealed low brain volumes in the anterior temporal lobe and frontal lobes.

**Figure 2:**
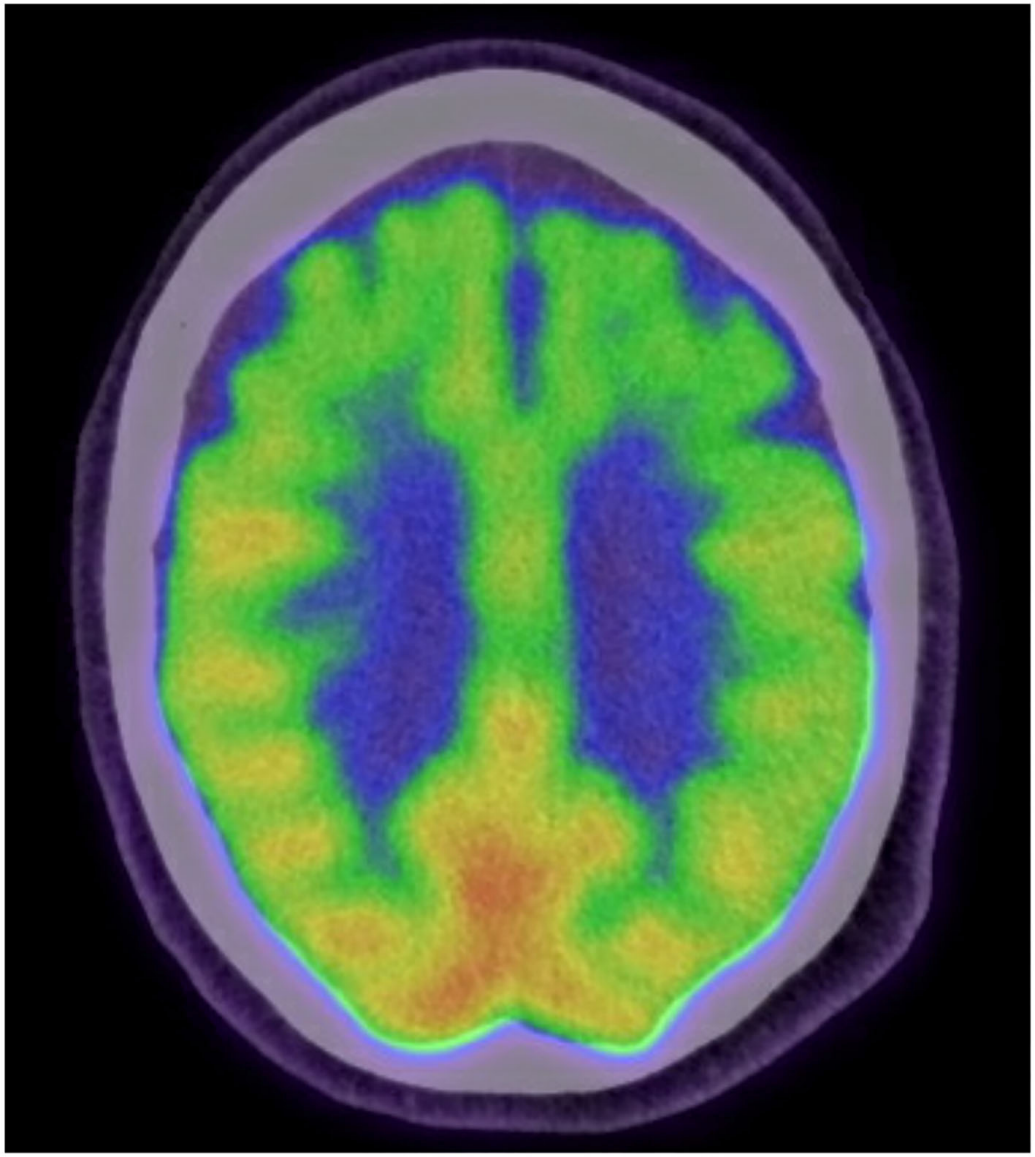
The patient's fluorodeoxyglucose (FDG)-positron emission tomography scan showing hypometabolic areas primarily in the left temporal lobe and biltateral frontal lobes.

An 84-year-old right handed, white male presented to our clinic with a 1 year history of speech changes. He had been initially diagnosed with “an invisible stroke” by a general practitioner, however, when symptoms continued to progress, he was referred for tertiary consultation. On initial examination he exhibited the hallmark features of nfvPPA, most notably, speech with short ungrammatical sentences that included nouns but omitted verbs, adverbs, and adjectives. Formal neuropsychological evaluation revealed extremely low verbal fluency, poor repetition, and mildly impaired naming ability with occasional paraphasic errors. Performance on executive function tasks was mostly within normal range with the exception of difficulty on a task of visuomotor set shifting (Trails B). Tests of memory, attention, and visuospatial functioning were normal. Other than a slight slowing of saccade initiation and upward gaze, his neurological examination was generally unremarkable and free of Parkinsonism, scoring a 2 (for a postural tremor and gait) on Part III of the Unified Parkinson's Disease Rating Scale (UPDRS). In regards to neuropsychiatric status, he admitted to being very frustrated by his speech difficulties but he denied symptoms of depression or anxiety and denied suicidal ideation. He scored a 0 on the Geriatric Depression Scale (GDS). The results of his magnetic resonance imaging (MRI) revealed atrophy of the midbrain, diffuse regions of the frontal lobes, and focal atrophy of the anterior pole of the left temporal lobe (Figure 1). A fluorodeoxyglucose (FDG)-positron emission tomography scan showed left temporal lobe hypometabolism and supported the diagnosis of nfvPPA (Figure 2). Of note, there was no family history of dementia and a targeted genetic panel (Athena Diagnostics Inc.) failed to reveal mutations in the progranulin gene (PGN), microtubule associated protein tau gene (MAPT), or expansions of the C9orf72 gene.

Over the course of the next 3 years the patient experienced a slow and steady decline in language. Like many patients with nfvPPA he also started to experience a marked decline in motoric functioning including several backwards falls, eye movement difficulties, and slowed gait. From a behavioral perspective he became more apathetic, and, per his wife's report, impatient and irritable, including throwing things when he did not get his way. He also grew increasingly frustrated with his speech and intermittently refused to take part in some family outings. Despite these motor and behavioral changes, his general cognition and functioning remained at a mild dementia level as he continued to manage his own medications and do odd jobs around the house (Global Clinical Dementia Rating = 1). Although he was aware and concerned about his speech he continued to deny depression, suicidal ideation, or anxiety on any of the standard screening measures (GDS) administered during his 6 month clinical assessments. Because of his irritability, he was started on escitalopram which was gradually titrated to a max dose of 20 mg/daily with his wife noting minimal improvements in mood and anxiety. A former Marine and veteran of the Vietnam War, the patient admitted to owning a handgun “for protection” that he kept locked and loaded in a safe in his garage.

His suicide came as quite a shock to both his clinical team and family. His wife could not identify any preceding triggers or changes in his mood or behavior. He appeared more future-oriented than ever, looking forward to a family fishing trip planned for the following week. On the day of his suicide he had been relaxing comfortably as he did most afternoons. He intermittently went into the garage to work or clean his tools. During one of his trips to the garage he took his life with a single gunshot to the head using the handgun that was stored in his locked safe. He did not leave a note or say goodbye to his wife, family, or friends.

## Discussion

Increasing age is a risk factor for both dementia and suicide and there is increasing body of research suggesting that individuals with non-AD dementia may be at particularly high risk (16–18, 21). To our knowledge this is the first case report of completed suicide in an individual diagnosed with nfvPPA. This patient had several clearly identifiable demographic risk factors for suicide in the elderly: white race, male sex, medical disability, cognitive decline, military veteran, and access to firearms and ammunition (23). There is less data regarding how FTD contributed to his risk of suicide. Several small studies have shown that suicide risk might be significantly higher in FTD (16, 17). Characteristic phenotypic differences between AD and FTD have been used to explain this risk including preserved insight, higher neuropsychiatric burden, and, on average, a significantly younger age of onset (younger age may be associated with less oversight, functional impairment, and increased ability to carry out suicide). Retained insight, in particular, seems to be a key risk factor for suicide in all forms of dementia and the primary reason suicide risk seems to be highest in the first year after a diagnosis (24). Individuals with PPA have better insight which may manifest as frustration, depression, and hopelessness as individuals increasingly struggle to communicate (25).

Associations between completed suicide and dysfunctional glutamate systems have been reported in several cognition-associated brain regions including the dorsolateral prefrontal cortex, orbitofrontal cortex, and cingulate cortex (26, 27). Interestingly, these are the typical brain regions that undergo early and significant atrophy FTD. Although dysfunction in multiple neurotransmitter systems, most notably serotonin and dopamine, have been commonly implicated in the pathogenesis of FTD, there has been a renewed emphasis on the role of glutamate dysfunction in FTD (28). Memantine is an N-methyl-D-asparate (NMDA) receptor antagonist that modulates aberrant NMDA activity and preserve normal synaptically released glutamate (29). Memantine has been studied in two clinical trials in FTD, both of which failed to meet cognitive outcomes (30, 31, 43). It is, however, used “off-label” to treat cognition and/or behavioral disturbances in individuals with FTD (32). This patient was not being treated with memantine at the time of his suicide.

Depression and affective disorders are among the strongest risk factors for suicide (32). A unique aspect of the case presented above is that suicide did not appear to be preceded by an affective/depressive prodrome. In fact, he neither “flagged” on his depression screener nor did he present with the mental status changes that one would typically associate with depression. At each appointment he denied suicidal ideation (assessed through a standard question during each clinical visit). The outcome in this case suggests that screening for depressive symptoms may not be sufficient enough to predict suicidal behavior in individuals with FTD.

Current research framework for suicide includes a prototypical chronology of ideation, planning, and attempt (11). It is therefore, notable to mention that this case did not follow the typical chronology of suicidal behavior. In retrospect, his suicidal act appeared rather impulsive in nature and also coincided with the emergence of impulsivity in other areas of life (losing temper, throwing things, etc.). Impulsivity is an important, but often overlooked predictor of suicidal behavior across multiple psychiatric diagnoses (33, 34). Impulsivity has also been associated with reduced gray matter volumes across multiple frontal regions in individuals with borderline personality disorder (35). Given that impulsivity and frontal brain atrophy is a hallmark feature of FTD, this case may suggest that the emergence of impulsivity could be an important risk factor for suicide in FTD and may be useful in predicting individuals at increased risk for suicidal behavior.

Finally, this case demonstrates the importance of addressing gun safety in individuals with dementia. Those over the age of 65 now have the highest rate of gun ownership in the United States (36). It is estimated that up to 50% of older individuals live in a home with a gun (37), but there has been little research and few guidelines around gun ownership in patients with dementia. Only two states have laws specifically addressing the issue of gun ownership by persons with dementia. Studies have shown although families of individuals with dementia report that they prefer to discuss gun safety with their medical providers, a very small percentage have ever discussed it with a medical provider (38). Given high rates of case fatality with firearms in the elderly, especially among men over the age of 80 (who have a suicide rate more than double the national average) (39), this represents an important gap in geriatric medicine. At the very least, it is imperative that health care providers routinely address firearm safety as they would other safety concerns such as driving. An approach for inquiring about gun safety is the 5 L's (40). The first question is to simply ask “Is there a firearm in the home?” If the presence of a gun is confirmed, more information is gathered around the 5 L's: 1. Is it **L**oaded? 2. Is it **L**ocked? 3. Are **L**ittle children present? 4. Is the operator feeling **L**ow? 5. Is the operator **L**earned? An affirmative answer to any one of the 5 L's should result in further action in accordance with family support and state law. Simple steps such as keeping a gun locked and unloaded and storing ammunition in a separated locked area has been associated with fewer self-inflicted deaths (41). A recently published algorithmic approach to firearm safety in cognitive impairment provides guidance based on an individual's functional status (42). Applying this approach may have been helpful in this case and warrants further testing in prospective studies.

## Data Availability Statement

The original contributions presented in the study are included in the article/supplementary material, further inquiries can be directed to the corresponding author/s.

## Ethics Statement

Ethical review and approval was not required for the study on human participants in accordance with the local legislation and institutional requirements. Written informed consent from the patient was not required to participate in this study in accordance with the national legislation and the institutional requirements. Written informed consent was obtained from the next of kin for the publication of any potentially identifiable images or data included in this article.

## Author Contributions

DP was a major contributor in reviewing the literature and writing the manuscript in all parts aside from the case itself. SA contributed to review of the literature as well as writing and editing. KS helped with writing, was an editor and assembled documents necessary for submission. AR provided the case report and guidance to completion. All authors read and approved the final manuscript.

## Funding

This work was supported by P20 GM109025-06A1 grant.

## Conflict of Interest

The authors declare that the research was conducted in the absence of any commercial or financial relationships that could be construed as a potential conflict of interest.

## Publisher's Note

All claims expressed in this article are solely those of the authors and do not necessarily represent those of their affiliated organizations, or those of the publisher, the editors and the reviewers. Any product that may be evaluated in this article, or claim that may be made by its manufacturer, is not guaranteed or endorsed by the publisher.
